# The Effect of Moment of Inertia on the Liquids in Centrifugal Microfluidics

**DOI:** 10.3390/mi7120215

**Published:** 2016-12-02

**Authors:** Esmail Pishbin, Manouchehr Eghbal, Sepideh Fakhari, Amin Kazemzadeh, Mehdi Navidbakhsh

**Affiliations:** 1 Mechanical Engineering Department, Iran University of Science and Technology, 1684613114 Tehran, Iran; es.pishbin@gmail.com (E.P.); fakhari_s@mecheng.iust.ac.ir (S.F.); mnavid@iust.ac.ir (M.N.); 2 Lab-on-a-disc Technology Center (LTC), Iranian Research Organization for Science and Technology (IROST), Ahmadabad Mostoufi, Azadegan Highway, 3353136846 Tehran, Iran; 3 Science for Life Laboratory, Division of Nanobiotechnology, KTH Royal Institute of Technology, SE-100 44 Stockholm, Sweden; kewmars.k.zad@gmail.com

**Keywords:** lab-on-a-chip, microfluidics, inertia, disc, pump, mixing

## Abstract

The flow of liquids in centrifugal microfluidics is unidirectional and dominated by centrifugal and Coriolis forces (i.e., effective only at T-junctions). Developing mechanisms and discovering efficient techniques to propel liquids in any direction other than the direction of the centrifugal force has been the subject of a large number of studies. The capillary force attained by specific surface treatments, pneumatic energy, active and passive flow reciprocation and Euler force have been previously introduced in order to manipulate the liquid flow and push it against the centrifugal force. Here, as a new method, the moment of inertia of the liquid inside a chamber in a centrifugal microfluidic platform is employed to manipulate the flow and propel the liquid passively towards the disc center. Furthermore, the effect of the moment of inertia on the liquid in a rectangular chamber is evaluated, both in theory and experiments, and the optimum geometry is defined. As an application of the introduced method, the moment of inertia of the liquid is used in order to mix two different dyed deionized (DI) waters; the mixing efficiency is evaluated and compared to similar mixing techniques. The results show the potential of the presented method for pumping liquids radially inward with relatively high flow rates (up to 23 mm^3^/s) and also efficient mixing in centrifugal microfluidic platforms.

## 1. Introduction

Since the advent of microfluidic technology, the miniaturization, integration and automation of clinical and biochemical processes have engaged multiple researchers around the world [1]. Clinical processes often entail various time-consuming and laborious procedures such as sample preparation, introducing various reagents, and detection that requires skilled operators and centralized laboratories [2]. Lab-on-chip (LOC) is an inexpensive technology that has been practiced in several international research groups in order to integrate, miniaturize, and automate these processes [3]. Despite the availability of a few commercialized LOC devices, e.g., I-stat [4], LOC platforms usually fail to successfully integrate and automate clinical diagnostics which require several steps of sample preparation, polymerase chain reaction (PCR), culturing, etc. Lab-on-disc is a sub-set of micro total analysis systems (μTAS) technology that exploits inherent forces on rotary disc–shaped platforms to control the liquid flow using minimal external power sources [5,6]. These features allow for a step forward towards the realization of the concept of clinical point of care (POC) devices by enabling the on-chip preparation of samples.

The effects of the inherent forces on the centrifugal microfluidics (centrifugal, Coriolis, and Euler) on the liquid are the basics for designing of the various functional elements, such as pumping or valving, in the microfluidic discs. In order to efficiently exploit the disc real estate, it is necessary to develop novel flow manipulation techniques to push the liquid against these inherent forces. The clinical assays often consist of numbers of non-linear tasks that are performed on centrifugal microfluidics where the liquid flow remains centrifugally outward and unavoidably occupies the usable radial space [7].

Flow manipulation techniques can be categorized into two main types, passive and active methods. Several significant passive techniques such as the elastic deformation of rubber-like materials and active methods such as most pneumatic–based mechanisms have been developed to conduct different microfluidic tasks, i.e., mixing, aliquoting, siphoning, flow switching, etc. [8,9,10,11]. Various siphon-based techniques usually employ the capillary forces, and recently Euler force, to transport liquids in short-distance ranges toward the center basically for siphon priming [12,13]. Since the lab-on-disc materials are usually hydrophobic, siphoning needs special surface modifications to allow for capillary action. The latex micro-balloon that uses the potential energy stored in the elastic latex material to pump fluids towards the disc center [14] and pneumatic pumping methods which use the energy stored in the compressed trapped air [15,16] are some recent examples of passive pumping where the pneumatic methods consume additional disc space to trap and compress the air. The latex micro-balloon technique is a significant improvement and appears to be highly efficient for mixing different liquids; however, the integration of latex layers with the polymeric membranes imposes additional fabrication steps which can be more difficult, particularly when a fabrication method other than machining is used.

The centrifugo-dynamic pumping mechanism is a well-known technique in the passive pneumatic-based mechanisms which employs the pneumatic energy of compressed air in a ventless chamber for pushing liquids toward the center. Although numerous elements with relatively complex geometries are required for the microfluidic design of the structure, the efficiency of the method is relatively high (>75%) and there is no assistive equipment for the production of compressed air, unlike most other pneumatic techniques [17]. Furthermore, this method has been utilized for applications such as aliquoting [9] and valving [18]. There are also some methods based on liquid suction in microchannels using a secondary liquid for pulling/pushing the intended liquid toward the disc center [19,20].

Active methods use an external power source such as heaters [21] or compressors [20] to pump liquids. For example, Abi-Samra and colleagues used an infrared lamp to heat the air in a ventless chamber and used the energy produced to push the liquid towards the disc center [20]. Employing a pressure difference between the chamber containing the sample/reagent and a secondary fluid to pull the liquid towards the disc center has also been reported recently [21]. In this method, the liquid is pulled and pushed during exothermic and endothermic processes, respectively. Although active methods allow for more complex processes, they need external equipment that increases the size and weight of the whole system, and may not be an appropriate approach for POC devices [16].

We presented a novel method for moving the flow centrifugally inward in a passive and simple way. The technique is based on the moment of inertia of the liquid enclosed in a chamber on a rotating microfluidic disc. The tendency of the liquid to maintain the rotational velocity of the disc is employed to transfer the liquid from the source chamber to a destination chamber located radially closer to the disc center. The effect of the liquid moment of inertia on its motion is theoretically and experimentally discussed and an application of the moment of inertia is presented.

## 2. Methodology

### 2.1. Concept

Forces other than the centrifugal force, e.g., Coriolis and Euler, are not adequate in magnitude to be used for guiding large volumes of liquids (in the scale of mm^3^) towards the disc center. However, such forces can be employed to influence the direction of the flow, e.g., the use of Euler force for enhancing the siphoning mechanism [13]. For the first time, we used the inertial force of a liquid enclosed in a chamber in centrifugal microfluidics, rotating with a given rotational velocity, for pushing the liquid into a microchannel connected to a chamber near the center of the disc. The liquid in a chamber can be assumed as a rigid body having the same angular momentum to its equivalent portion of the disc. When the disc experiences a rapid reduction of the rotational velocity, the liquid responds to the new circumstance and will be defined as a continuous motion (for a limited time) in the direction tangent to the rotational velocity vector of the disc. This reacting flow can be directed to a destination chamber often located radially closer to the disc center via a microchannel. The whole or partial volume of the liquid can be transferred to the destination chamber if the pseudo-force acting on the liquid overcomes the pressure losses occurring during the displacement and the surface tension forces. This allows for pushing of the liquids to a different chamber without using an external power source or without introducing intricacy to the system.

**Figure 1:**
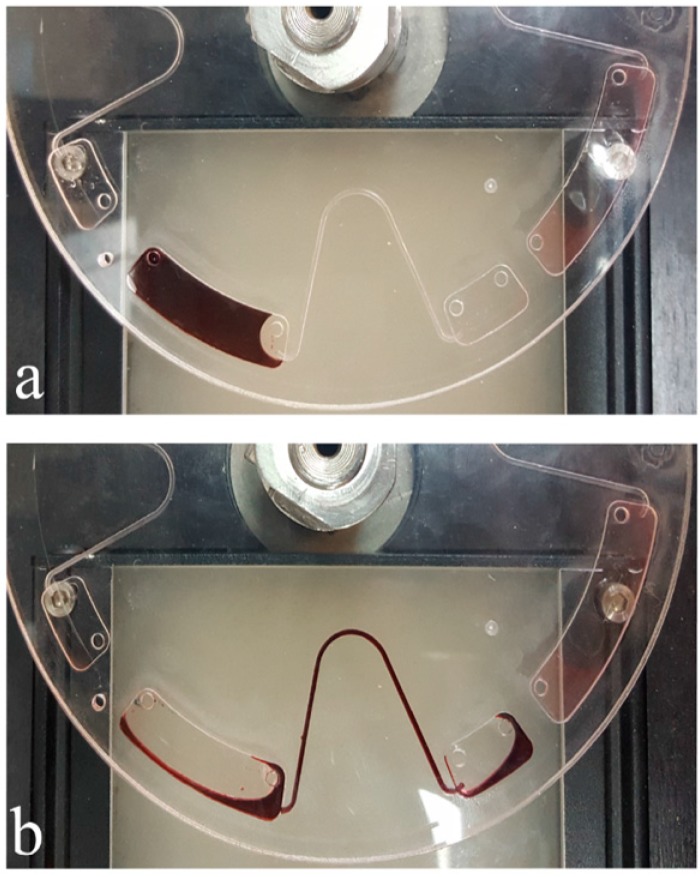
The top view of a centrifugal microfluidic disc; (**a**) the liquid is pipetted into the chamber and the disc is spun at 2500 RPM (C.C.W); (**b**) the rotational velocity of the disc is abruptly decreased to 50 RPM and the liquid entered the next chamber.

Figure 1 demonstrates an experiment design for evaluating the concept presented. The designed microfluidic disc consists of two vented chambers located near the edge of the disc which are connected by a microchannel that passes near the disc center. Then, 150 μL of a dyed liquid with such properties as water has been pipetted into the primary chamber. Afterward, the rotational velocity of the disc reached 2500 RPM and after a while, followed by an abrupt deceleration (3000 RPM/s), reached 50 RPM. The results show that a considerable volume of liquid is transferred to the destination chamber. The amount of transferred liquid in this condition is approximately 65 μL in the first cycle and 30 μL in the second cycle in the same conditions.

### 2.2. Experimental Setup and Fabrication

A fully customized platform (CD Imager K1000, Key Lead Solutions Inc., San Francisco, CA, USA) that has been specifically designed for image capturing on a spinning disc was used in our study. For the disc design evaluation, the motion of deionized (DI) water dyed with food dyes in the chamber was observed utilizing an integrated disc driver and a strobe lighting system for image capturing. The driver unit is able to apply specific spinning frequencies and acceleration/declaration to the disc, using a brushless servo motor. It is equipped with a fully programmable corresponding motor controller for high precision movement. The imaging section of the machine consists of a stereo microscope, a mounted high-speed camera (1.4 Mega Pixel CCD camera and maximum frame rate of 120 fps) and a strobe light with a fiber optic system. The system employs a special corresponding software to export the images for further analysis.

The centrifugal microfluidics consists of layers of Polymethyl methacrylate (PMMA) that are bonded together using pressure sensitive adhesive (PSA) layers. The centrifugal microfluidics were specially designed using a commercial computer aided design (CAD) software. The 1 mm thick PMMA (Chochen, Tainan, Taiwan) substrates were cut by a computer numerical control (CNC) machine (Roland, Japan—mdx-40A) according to the CAD designs. The designs comprised of the source and destination chambers connected by a microchannels. The 100-μm-thick and optically clear PSA (DFM 200 clear 150 POLY H-9V-95, FLEXcon, Spencer, MA, USA) were cut to exactly conform to the PMMA designs using a cutter plotter (Graphtec, Japan—Graphtec CE-6000). The PMMA layers were washed, sterilized using ethyl rubbing alcohol, and dried using an air compressor. The PMMA and PSA layers were aligned and sandwiched using a screw press to form the centrifugal microfluidics especially designed for demonstrating a novel liquid handling and mixing technique.

## 3. Results and Discussions

### 3.1. Characterization

**Figure 2:**
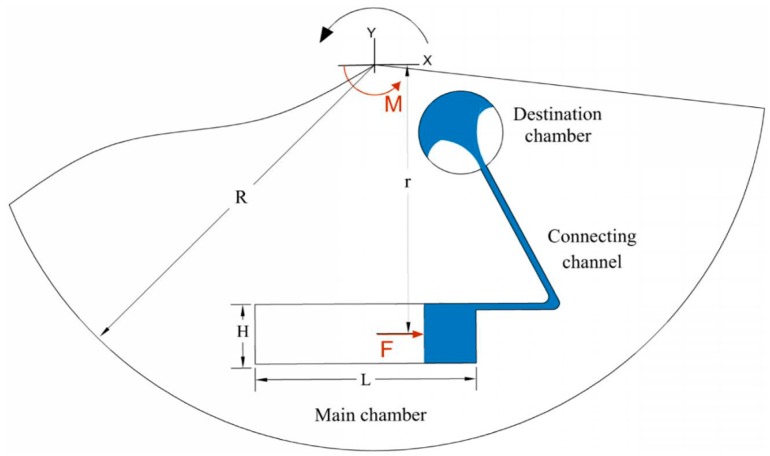
The schematic view of showing the effective parameters, the microchannel connecting the source and the destination chamber.

We assumed that the disc was rotating with the rotational velocity of ω, and due to the artificial gravity forces applied to the system, the liquid in the chamber behaved as a rigid body. The moment of inertia of the liquid due to the rapid reduction of the rotational velocity was calculated as:

(1) M=Iα

where *M*, *I*, α are the moment of inertia which is applied in the center of the disc, the mass moment of inertia of the liquid and the rotational deceleration of the disc, respectively. Assuming the liquid in the chamber as a rectangular block, the value of the liquid mass moment of inertia about the *z*-axis (shown in Figure 1) in the center of the disc is [22]:

(2) $$I=\frac{1}{12}m\left(H^{2}+L^{2}\right)+mr^{2}$$

where *L*, *H*, and *m* are the length, the height, and the liquid total mass of the rectangular block, respectively, and *r* is the vertical distance between the *x*-axis passing through the liquid mass center and the center of the disc (Figure 2). According to Equation (2), the mass moment of inertia is a function of the length, the height, and the mass of the liquid. The force applied to the liquid due to this moment of inertia can be approximately calculated by assuming the liquid is acting as a rigid body. Since the pressure of the liquid in the inlet of the connecting channel and destination chamber is equal to the ambient pressure, the applied pressure on the liquid (*P*) can be calculated as:

(3) $$P=\frac{I\alpha }{rs}=\frac{\left[\frac{1}{12}m\left(H^{2}+L^{2}\right)+mr^{2}\right]\alpha }{rHs}=\frac{m\alpha }{rs}\left[\frac{1}{12}\left(H+\frac{L^{2}}{H}\right)+\frac{r^{2}}{H}\right]$$

where *s* is the thickness of the liquid chamber. In order to efficiently transfer/evacuate liquid from the source chamber to the destination chamber, the geometrical parameters of the source chamber have to be optimized. We assumed that the liquid is incompressible with a given mass (*m*) and distance from the disc center (*r*), the thickness of the chamber is fixed, and therefore, and the height (*H*) and the length (*L*) of the source chamber are the only geometrical parameters that affect the applied pressure value. Considering that:

(4) $$j=\left[\frac{1}{12}\left(H+\frac{L^{2}}{H}\right)+\frac{r^{2}}{H}\right]$$

For maximizing the applied pressure value, the parameter *j*, the variable geometrical part of Equation (3), must be at its maximum. Therefore, the optimal values of the *H* and *L* could be calculated using Equation (4) assuming that (length) × (height) and also the chamber position (*r*) are constant. The optimized geometrical parameters were calculated as:

(5) $$L_{\mathrm{opt}}={\left(-r^{2}+\sqrt{12r^{4}+C^{2}}\right)}^{\frac{1}{2}}\text{ and }H_{\mathrm{opt}}=\frac{C}{{\left(-r^{2}+\sqrt{12r^{4}+C^{2}}\right)}^{\frac{1}{2}}}$$

where: *C* = *H*·*L* = *V*/*s* (a constant value). Note that Equation (5) has been derived for a rectangular shape of liquid in a chamber. The same principle can be used to derive the optimal values for shapes other than a rectangle. In addition, the time required for the liquid displacement through a microchannel to the destination chamber is reversely proportional to the deceleration of the disc, and the volume flow rate of the liquid is also directly proportional to the rate (time) of reducing the angular velocity. This time can be calculated using the relation between the rotational velocity and the acceleration:

(6) $$\Delta t=\frac{\Delta \omega }{\alpha }$$

The transferred volume of the liquid during one cycle of the moment of inertia method can be calculated by the Poiseuille equation and the total operation time. Since the final equation for the transferred liquid for an optimized shape of the chamber is:

(7) $$V_{\mathrm{transfered}}=\frac{\pi \rho D_{h}^{4}}{128µL_{c}\Delta \omega }\left[\frac{L_{\mathrm{opt}}}{r}\left(\frac{1}{12}\left(H_{\mathrm{opt}}^{2}+L_{\mathrm{opt}}^{2}\right)+r^{2}\right)\right]$$

For simplification, the head losses in the microchannel inlet and outlet and the surface tension forces were neglected and the pressure value was assumed to not be time-dependent in the pumping operation. Equations (6) and (7) imply that there is an optimal value of deceleration that limits the volume of the liquid that can be transferred by a certain applied pressure.

### 3.2. Exprimental Validation

For validating the derived equations results, we designed an experiment for comparing the theoretical and experimental values of the transferred liquid volumes using the moment of inertia method. In this design, the volume of the main chamber was 80 μL, and so the optimum length and height were calculated for the chamber based on Equation (5) (24.8 mm and 3.22 mm, respectively). The average distance of the center of mass of the chamber from the disc center (*r*) was selected as 50 mm. The chambers were connected by a 2-mm-long microchannel. The hydraulic diameter of the microchannel was 0.7 mm. The disc was accelerated up to 2000 RPM and then an abrupt deceleration (2000 RPM/s) was applied. The approximate volume of the liquid transport to the destination chamber in the first cycle was 23.7 μL (the pumping efficiency was 30%). Implementing Equation (7) and using the height and length values of the designed chamber resulted in 25.52 μL. The small differences of the theoretical and experimental values could be due to neglecting the effect of the centrifugal force on the liquid flow rates.

The performance of the pumping based on the moment of inertia method could be compared with previously presented techniques such as the centrifugo-dynamic method [15]. This method is a passive pneumatic-based technique that seems to be one of the most applicable methods among the liquids pumping in centrifugal microfluidic platforms. The maximum pump efficiency of the method has been reported at about 75% for liquid volumes between 200 and 300 μL at a deceleration of 30 Hz (1800 RPM). Obviously, for greater liquid volumes the efficiency decreases. As a comparison, the functional deceleration for both methods is approximately the same; however, it seems that for higher requirement volumes of the pumped liquid and flow rates, the moment of inertia method works better, while the pneumatic-based method is suitable for liquid volumes less than 300 μL. Furthermore, there is more space required for the centrifugo-dynamic method because of the use of an extra chamber for generating the compressed air as the key element for pumping the liquid, while in the current method, pumping can be performed in simple structures that are connected to a microchannel and there is also no requirement for small channels.

The relatively high deceleration values for the liquid pumping function based on the presented method cause the cost of the processing device for performing such acceleration rates to increase. Further studies are necessary for geometrical solutions, such as the method that Schwemmer et al. presented for the centrifuge-pneumatic mechanism using a timer with a small channel that decouples the pneumatic pumping from high deceleration rates [23]. Furthermore, special surface modifications could be suggested for transporting more volumes of samples in a more efficient way.

## 4. Application

### 4.1. Mixing Concept

The mixing of various biochemical and biomedical reagents is an essential task in almost all clinical diagnostic procedures. As an application of our novel flow-handling technique, we demonstrate the mixing process of two differently dyed DI water. Despite the fact that in other microfluidic approaches, mixing is reliant on diffusion techniques and is usually applicable, it is not suitable in centrifugal microfluidics due to the relatively higher liquid volumes used in such devices [24]. Therefore, the majority of the mixer elements reported on the centrifugal microfluidics use a convective type of mixing [24,25,26]. It is necessary for operations such as the polymerase chain reaction (PCR) due to the small sizes of DNA molecules, and the diffusivity number. However, achieving a homogenous mixture is a challenging task in centrifugal microfluidics due to the laminar nature of the flow [27]. The two main types of mixing methods can be defined as passive which does not require external power sources and active which does often require external power sources. In active convective mixing, the liquid streams of interest often flow separately and combine in a secondary chamber. For instance, the batch-mode mixing mechanism mixes different reagents by alternatively changing the rotational velocity and also by utilizing the magnetic beads’ motion in the liquid [28]. In addition, the reagents can be split and recombined several times in order to achieve a uniform mixture [29]. The forces to split and recombine the liquids are provided from a thermal or a thromopnumatic source that imposes an additional cost/complexity to the system. In the passive mixing methods, the liquids of interest usually flow through specifically designed microchannels such as a serpentine channel [30]. Using bubbles, generated by the buoyancy effect, for mixing is a newly presented technique that leads to the production of a homogenously mixed liquid in a fixed rotational velocity [31]. These specific microstructures often require a large area of the disc and therefore limit the number of operations that can be conducted per disc. Recently, a new mixing method has been introduced that uses the highly elastic properties of embedded layers of latex to mix different liquids in a single chamber [14].

However, the assembling and characterization of this method can be technically difficult and impose additional intricacy to the disc fabrication. For the first time, we used the moment of inertia of the liquid enclosed in a chamber to frequently transfer the intended liquids between two chambers without using any external power sources or costly surface treatments. We showed that the reciprocating motion of the liquids due to their moment of inertia provided by abrupt changes in the rotational velocity of the disc was able to efficiently mix the liquids in centrifugal microfluidics. Our approach combined the advantages of the batch-mode and the split and recombination techniques. Therefore, the overall mixing time was expected to be less than the batch mode and the number of reciprocations required for efficient mixing will also be less than that in the split-recombination method.

### 4.2. Mixing Design

**Figure 3:**
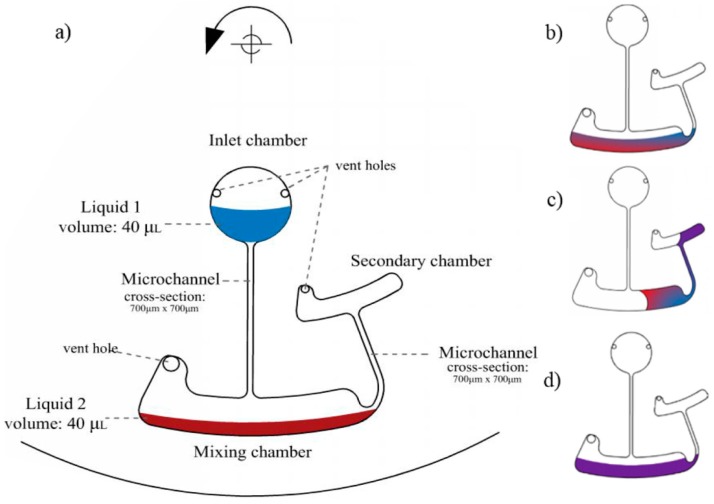
A schematic view of the mixing process and the mixing structure design; (**a**) the mixing components and the liquid volumes used in the experiments; (**b**) two different dyed liquids in the mixing chamber; (**c**,**d**) mixing cycle: the liquid flows into the secondary chamber at very low rotational velocity and flows back to the mixing chamber at higher rotational velocities.

**Figure 4:**
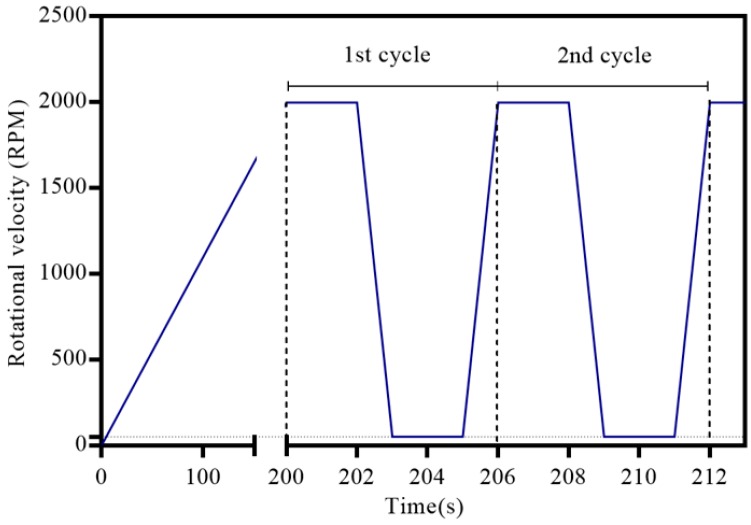
The rate of changes in rotational velocity of the disc during the mixing cycle (the disc is gradually spun to 2000 RPM).

A design consisting of two chambers containing two different liquids and a connecting microchannel was implemented for testing the mixing efficiency. Figure 3 shows the schematic design used for mixing in centrifugal microfluidics based on the moment of inertia method for two different dyed DI water. Figure 3a shows the positions and the dimensions of the chambers and the volumes of the different dyed DI water. The chamber length and height are approximately equal to their optimum calculated values based on Equation (5), 24.8 mm and 3.22 mm, respectively. Figure 4 shows the corresponding mixing cycle times and rotational velocities. The rotational velocity of the disc increases by 10 RPM/s in a counter-clockwise direction and the colorless DI water flows slightly towards the disc edge and enters the corresponding chamber at ~300 RPM. The colorless and the dyed DI water are united and compose a non-uniform mixture of colorless and dyed DI water. The rotational velocity was increased to 2000 RPM without observing any significant change in the mixing index of the mixture. Note that according to Equations (3) and (6), a rotational velocity and deceleration values of 2000 RPM and 2000 RPM/s, respectively, were required for pushing the liquid into a mixing chamber in 1 s. The rotational velocity of the disc was reduced to 50 RPM (i.e., the minimum RPM configuration of our running testing system) in a time of less than 1 s. A portion of the total liquid volume was transferred from the mixing chamber into a secondary chamber; the rotational velocity of the disc increased and the liquid flowed back into the mixing chamber.

### 4.3. Mixing Efficiency

**Figure 5:**
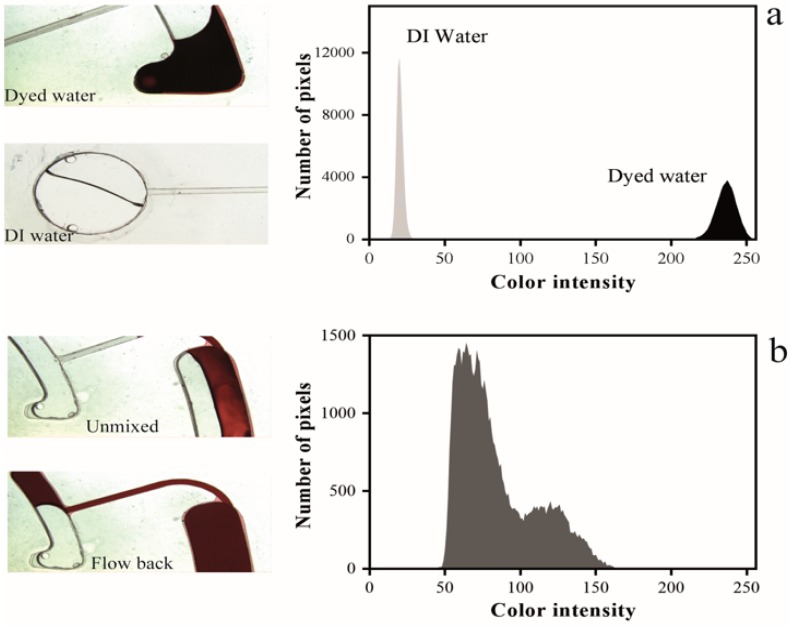

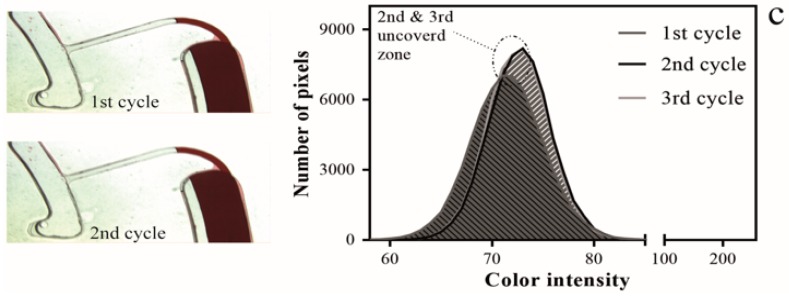
The color intensity histogram of mixing a colorless and dark red dyed DI water; (**a**) the color intensity of the DI water used in the experiment; (**b**) the color intensity of the liquids inside the mixing chamber before mixing; (**c**) the color intensity of the mixed liquid after first, second and third cycle.

The images of the liquid during the process were captured by a high-speed camera, and image processing was performed using a MatLab code for obtaining the color intensity histogram of the mixed liquid in various steps of the mixing process. Mixing based on the moment of inertia exploited the advantages of both the batch-mode and reciprocation techniques. The combination of the vortices generated due to the abrupt reduction of the velocity, the liquid flow in a narrow channel and the reverse motion of the liquid that takes place by increasing the rotational velocity significantly increased the mixing efficiency compared to other similar mixing methods. Figure 5 shows the color intensity histogram of the mixing process that was plotted using a MatLab code.

Specific regions of the obtained digital images of the pipetted and mixed liquids were analyzed. Each region contains 250 × 250 pixels and, using the histogram plot, the frequency of the pixel’s colors of the regions was plotted. The *x*-axis of the histogram graph represents the tonal variations of the red color and the *y*-axis represents the number of pixels in that particular tone. For measuring the dispersion of the data values, the standard deviation (δ), the square root of the variance, was used. The low values of this parameter show that the data points are near the mean value (higher mixing efficiency), and the high standard deviation values indicate that the data values are spread (lower mixing efficiency).

The figure also shows that a large portion of the mixing process occurred during the first cycle of the mixing. The second and third cycles had insignificant color intensity; repeating the process more than three times did not change the mixing index. This implied that the optimum mixing quality was achieved in the second cycle (standard deviation = 3.25). The standard deviation of the unmixed liquids was 25.5 which was reduced to 3.7 and 3.04 after the first and third mixing cycles. According to the small difference between the standard deviation values, it can be understood that the difference between the mixing index of the first, second and the third mixing cycles was approximately less than 2%. Note that we have assumed that the maximum mixing index was obtained after the third cycle due to the insignificant variation between the color intensity of the mixed liquids of the third and fourth cycles.

As a comparison of the moment of inertia mixing method to previously presented mixing methods, we operated the shake-mode mixing technique for the same liquid volume (40 μL of each), chamber geometries and dyed water color. The parameters of the rotation profile were adopted from Grumann et al. [28] (ω = 8 Hz (480 RPM), α = 32 Hz/s (1920 RPM/s) and *t* = 3 for rotational velocity, rotational acceleration/deceleration and time period of each cycle, respectively).

**Figure 6:**
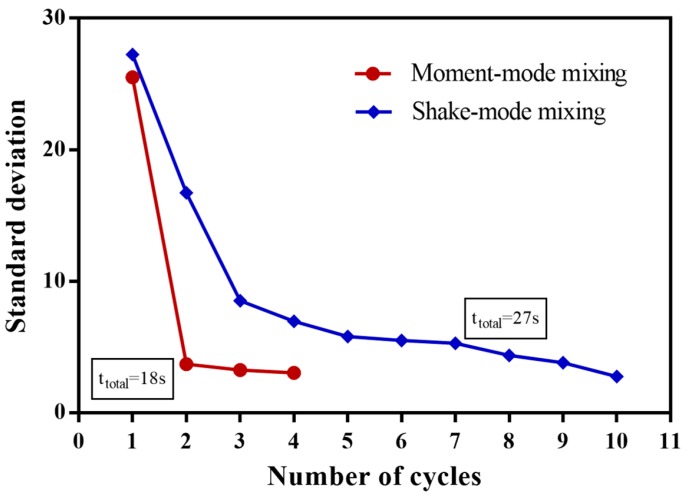
Demonstration of the variation of the histogram standard deviation value for the mixed liquid using shake-mode and moment of inertia mixing methods (the lower standard deviation value means higher mixing efficiency).

The standard deviation value of the histogram graph of the mixed liquids was calculated using the described image processing method after each rotation reversing. Figure 6 shows these values for the cycles of both the shake-mode and moment of inertia mixing methods. Presented results show that a relatively efficient mixing for the same primary mixed liquids can be achieved after three cycles (18 s) using the moment of inertia mixing method and nine cycles (27 s) using a simple shake-mode mixing technique.

Note that the geometry of the mixing chamber could influence the mixing results. We compared two mixing methods in the same conditions and chamber, which was geometrically optimized for mixing based on the moment of inertia method, to be able to compare the mixing methods while using other geometries for the mixing chamber, such as a circular-shaped chamber for instance, which could result in achieving shorter operational times for shake-mode mixing [28,31].

## 5. Conclusions

For the first time, the moment of inertia of the liquid was used to push the liquid towards the center of the microfluidic disc. The liquid enclosed in the chamber close to the disc edge was transferred and propelled towards the disc center by an abrupt reduction of the disc rotational velocity. The effect of the moment of inertia on the liquid in a rotational centrifugal microfluidic platform was theoretically and experimentally evaluated. It was shown that the maximum volume of the liquid transferred can be obtained by defining the optimum geometry of the chamber. The approach could be of value for mass production, etc., if small channel dimensions are critical. In addition, the moment of inertia effect was employed in order to mix two different dyed waters, and the mixing efficiency was evaluated by a MatLab code. The mixing technique was easy to implement based on the combination of the batch-mode and reciprocating technique. The mixing results and the method were compared to another mixing method which showed that a valuable mixing index is obtained in a shorter time when compared to shake-mode techniques.
